# A possible pathogenetic factor of sickle-cell disease based on fluorescent analysis via an optofluidic resonator

**DOI:** 10.1038/s41598-017-03634-8

**Published:** 2017-06-09

**Authors:** Hailang Dai, Cheng Yin, Xiaona Ye, Bei Jiang, Maowu Ran, Zhuangqi Cao, Xianfeng Chen

**Affiliations:** 1 The State Key Laboratory on Fiber Optic Local Area Communication Networks and Advanced Optical Communication Systems, Department of Physics and Astronomy, Shanghai JiaoTong University, Shanghai, 200240 China; 2 Collaborative Innovation Center of IFSA (CICIFSA), Shanghai Jiao Tong University, Shanghai, 200240 China; 3 Jiangsu Key Laboratory of Power Transmission and Distribution Equipment Technology, Hohai University, Changzhou, 213022 China; 4 Department of Physics and Electronic Science, Tongren University, Tongren, China

## Abstract

Waveguide based optofluidic resonator features high precision and high sensitivity in real-time fluorescent analysis. We present a novel optofluidic resonator following the hollow-core metal-cladding waveguide structure, which is then used to record the real-time binding process of Fe^2+^ and Fe^3+^ with protoporphyrin IX (PpIX) in PBS solution, respectively. The central fluorescent wavelength of compound with Fe^2+^ is in good accordance with that of the normal hemoglobin, whilst the peaks of the Fe^3+^ compound match the hemoglobin specimen from sickle-cell disease (SCD) patients. Similar statement holds when we monitor the real-time oxidation processes of these products by injecting oxygen into the optofluidic chip. These observations lead to the speculation that the SCD is caused by replacing the Fe^2+^ in hemoglobin with Fe^3+^, which may be insightful in the discovery of new clinical routes to cure this disease.

## Introduction

Sickle cell disease (SCD) occurs when the mutant sickle hemoglobin (Hb S) differs from the normal hemoglobin A by a single amino acid^1–5^. More than 300,000 children suffer from SCD in Africa every year^6, 7^, and the mortality is very high due to acute vaso-occlusive crises and increased risk of bacteremia^8, 9^. Although inexpensive interventions such as penicillin prophylaxis, vaccinations can be used to limit infection; and most of the childhood mortality could be avoided, if simple interventions and necessary diagnosis are accessible. Still more than 50% of children under 5 years of age die in low resource areas^10–13^. Many Studies analyzed the structural and physical properties of Hb S, which forms intracellular polymers upon deoxygenation. These investigations on SCD take place at the leading edge of the efforts devoted to elucidate the molecular basis of human disease. Pioneering studies by Pauling *et al*. established that SCD results from a defect in the hemoglobin molecule. Molecule fluorescent techniques have emerged as a powerful approach to describe bio-molecule structure, monitor reaction kinetics and detect molecule mutation. During the past decade, numerous applications based on the high-sensitivity fluorescent detection have been introduced, including DNA sequencing^14^, DNA fragment analysis^15^, fluorescence staining of gels following electrophoretic separations^16^, and a variety of fluorescence immunoassays^17^. Many present applications can be traced back to the early reports^18^, which has already anticipated them in advance. These efforts on the bio-molecule fluorescent are driven by the desire to eliminate the use of radioactive tracers, which are expensive to use and dispose of. However, the fluorescence technique has not received much attention in SCD clinicopathologic analysis. There is also an urgent need for rapid and low-cost testing methods, which are capable in a wide range of clinical, bioprocess, and environmental applications^19^. The purpose of this paper is twofold. First we illustrate the enhancement effect of the fluorescence spectra via a simple metallic waveguide structure by applying this technique to SCD. Second, we point out a possible clue that related with the SCD, which would be insightful in the searching of cure method. In our hypothesis, the heme in normal blood, which consists of Fe^2+^ and Protoporphyrin IX, can combine with and carry oxygen atom. In the contrast, Metheme exists in the blood of SCD patient and leads to the oxidized form of heme containing Fe^3+^ instead of Fe^2+^, which is incapable of binding oxygen.

The optofluidic resonator we used in this paper adopts the basic design of the symmetrical metal cladding optical waveguide structure^20^, where a fluidic chamber is inserted in the guiding layer. This kind of slab waveguide provides high-quality confinement to achieve efficient amplification of the radiation, which can be applied to enhance the fluorescence intensity. The quality factors Q, the spontaneous emission rate enhancement ration﻿ η of the hollow-core metal-cladding optofluidic resonator are detailed discussed in the supplementary information.Let us begin with the fluorescence spectrum of the hemoglobin specimen from the SCD patient and the healthy person. The Protoporphyrin, Hemoglobin and Sickle Hemoglobin provided by Ruijin Hospital (Informed consent was obtained from all subjects) are injected into the optofluidic resonator for fluorescent detection, and the concentrations of these samples are 10^−9^ g/ml. The structural parameters of the optofluidic resonator and the experimental setup will be described later.

**Figure 1:**
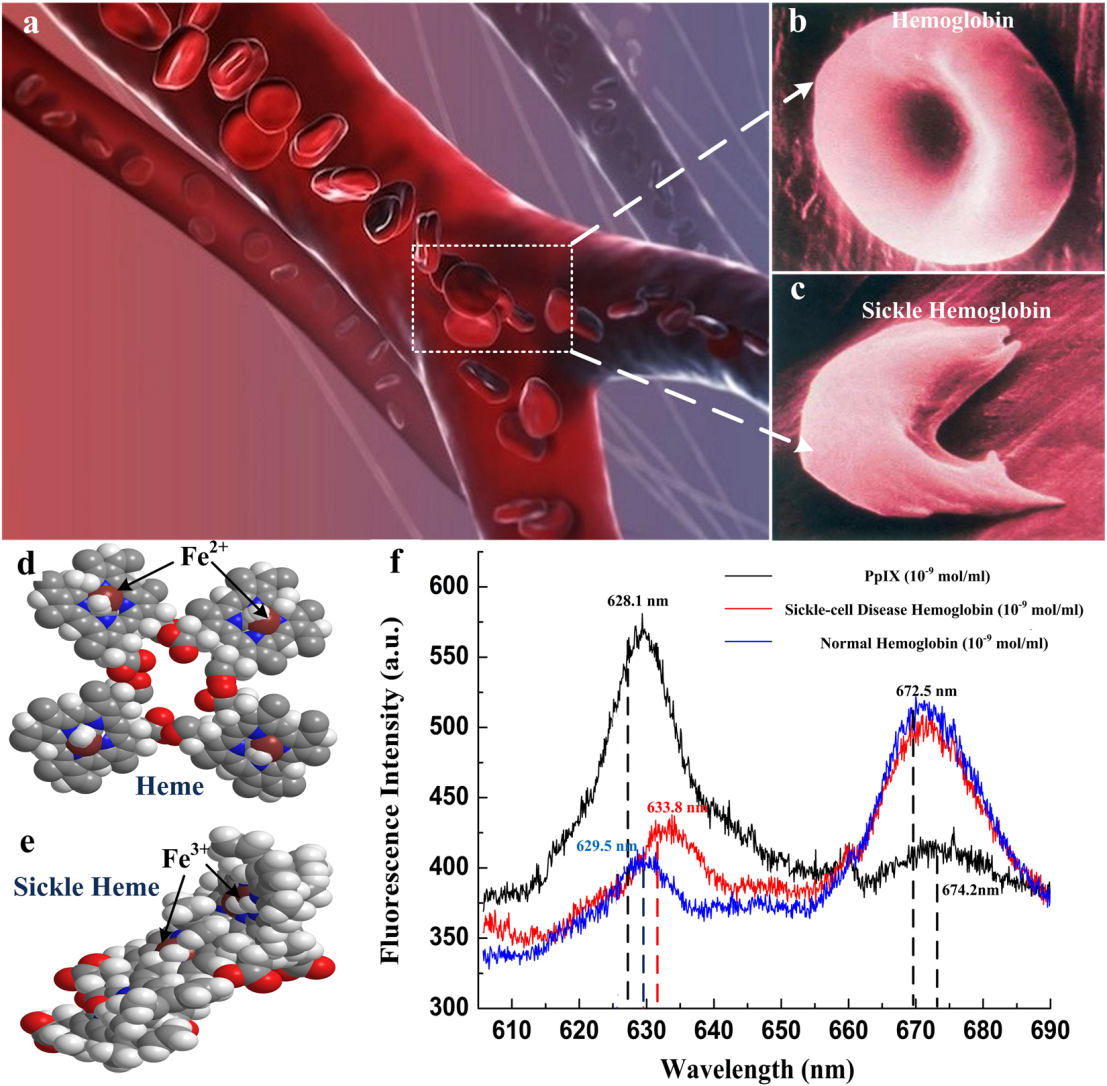
Hemoglobin from SCD patient and normal person. (**a**) SCD hemoglobin in vessels (drawn by 3ds Max 2013 soft ware); (**b**) and (**c**) Schematic diagrams of the normal Hemoglobin and SCD hemoglobin (drawn by 3ds Max 2013 soft ware); (**d**) and (**e**) Molecule models of the Heme and sickle heme (drawn by Chem 3D soft ware); (**f**) The fluorescent spectra of Protoporphyrin, Hemoglobin and Sickle Hemoglobin.

As can be seen from Fig. 1f, two fluorescent peaks with central wavelength at 628.10 nm and 674.20 nm appear in the fluorescent spectrum of Protoporphyrin, whilst the peak locates at 628.10 nm exhibits higher intensity. In comparison, Hemoglobin and Sickle Hemoglobin show different patterns that the fluorescent intensity at 672.50 nm appears higher. More important, when compared with Protoporphyrin, discernible shifts can be observed for the both left peaks, that the central wavelengths of the Hemoglobin and the Sickle Hemoglobin are shifted to 629.50 nm and 633.80 nm, respectively.

**Figure 2:**
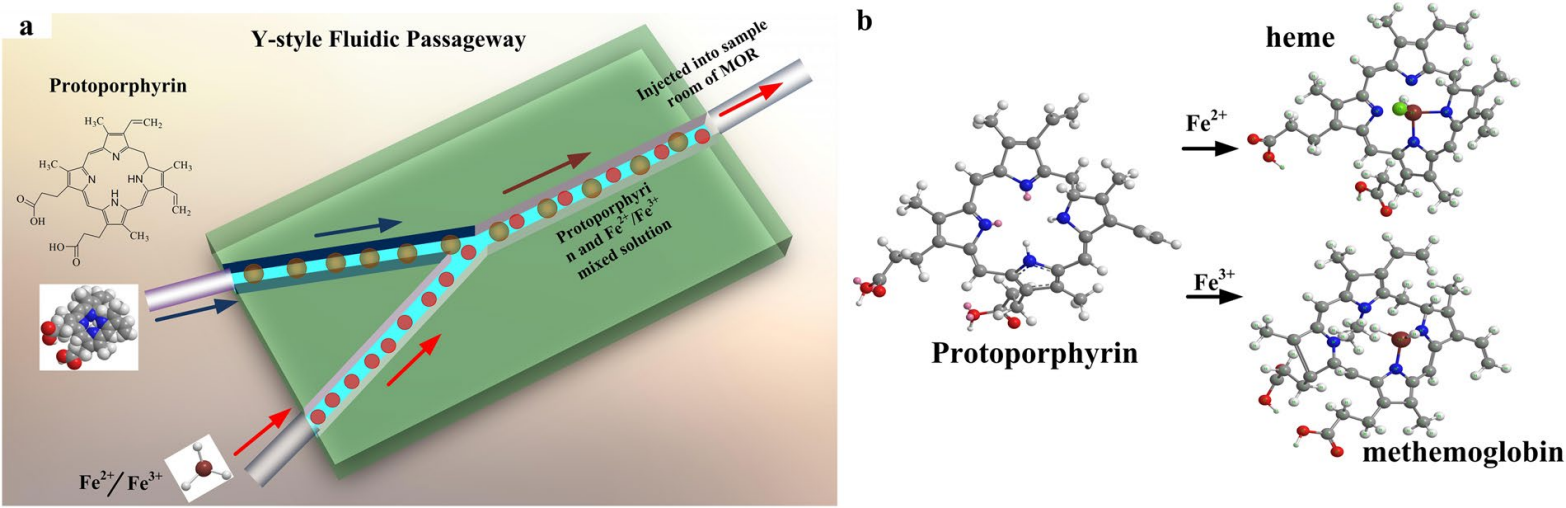
Y-style fluidic channel and molecules structure of metheme and heme. (**a**) The Y-style fluidic passageway (drawn by Solid Works 2012 soft ware); (**b**) The reaction process of Protoporphyrin IX with Fe^2+^ and Fe^3+^, respectively (drawn by Chem 3D software).

As shown in Fig 2b, the heme is combined by a Protoporphyrin IX molecule and Fe^2+^, while metheme is synthesized in the reaction with Fe^3+^. In our experiment, the Protoporphyrin IX (C_34_H_34_N_4_O_4_) is synthesized and dissolved in organic solvent N,N-Dimelthylformamide (C_3_H_7_NO) at room temperature of 20 °C. Fe^2+^ can be obtained when ferrous chloride (FeCl_2_·4H_2_O) is dissolved in deionizedwater. To avoid being oxidized, the solution should be kept in a weak acid environment (PH~6.3), and a trace of iron powder (0.01 mg) should be added. Furthermore, FeCl_3_ powder is dissolved in deionizedwater and to synthesize Fe^3+^, which is stable. All of the above mentioned solution are used immediately after they were ready.

The solutions are injected into sample room by intelligent trace syringes, and their mixing ratio can be controlled by the flow velocity of samples (Fig. 2a). In experiment, it is adequate to use the theoretical value for the approximate blending ratio 1:1. The Protoporphyrin IX, FeCl_2_ and FeCl_3_ solutions with low concentration of 10^−16^g/ml are used. If the concentration of Fe^2+^ or Fe^3+^ is much higher than Protoporphyrin IX, the excess iron ion will alter both the central wavelength and the fluorescence intensity^21^. On the other hand, the extremely low concentration of sample would result in weak fluorescent intensity, thus enhancement effect of the proposed resonator appears particularly important.

**Figure 3:**
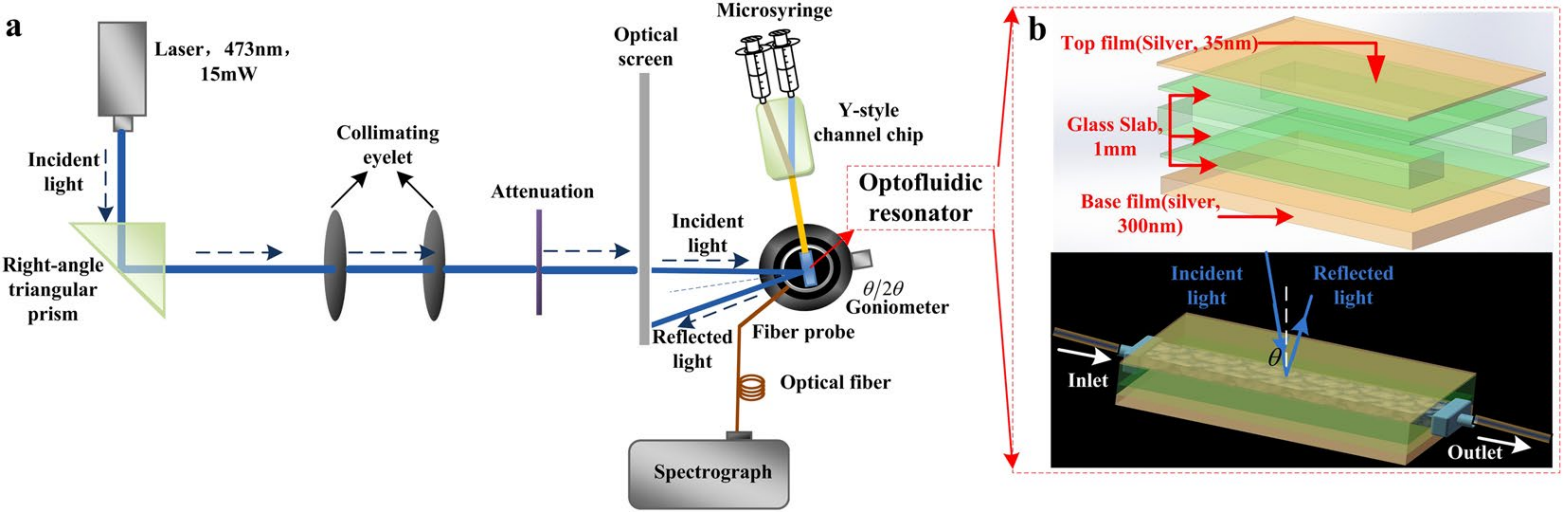
Experimental system and the optofluidic resonator. (**a**) The schematic image of the experimental setup (drawn by 3ds Max 2013 soft ware); (**b**) Structure of optofluidic resonator and the coupling light path (drawn by Solid Works 2012 soft ware).

The optical setup as shown in Fig. 3a is not complicate, due to the free space coupling technique. A computer controlled θ/2θ goniometer is applied for the accurate angular scan of the incidence to ensure the efficient energy coupling. The optofluidic resonator includes five layers, where the middle three layers form the guiding layer to support oscillating guided modes. From the top to the bottom, these five layers are a 35 nm Ag coupling layer, a 0.3 mm glass slab, a 0.5 mm sample layer, another 0.3 mm glass slab and a 300 nm Ag substrate. The size of the rectangular sample channel is 10 × 4 × 0.5 mm^3^. All these parts are optically contacted together with excellent parallelism. A spectrograph (Andor SR-750) is used to record the signal data collect by a 0.1 mm diameter fiber-optics probe, which locates close to the optofluidic resonator surface. The flow velocity is 10 μl/s, and the time interval of the recorded data is Δ*t* = 1*s*. Our strategy can be described as follows. First, fill the sample channel with Protoporphyrin, and excite an specific ultrahigh order modes (UOM) in the guiding layer by adjusting the incident angle to fulfill the phase match condition^22^. Second, the iron ion solution is injected into the sample channel to active reaction, while the fluorescence is significant enhanced due to the energy confinement and high sensitivity of the waveguide structure. Finally, the leaked fluorescence through the coupling layer is collected and recorded.

**Figure 4:**
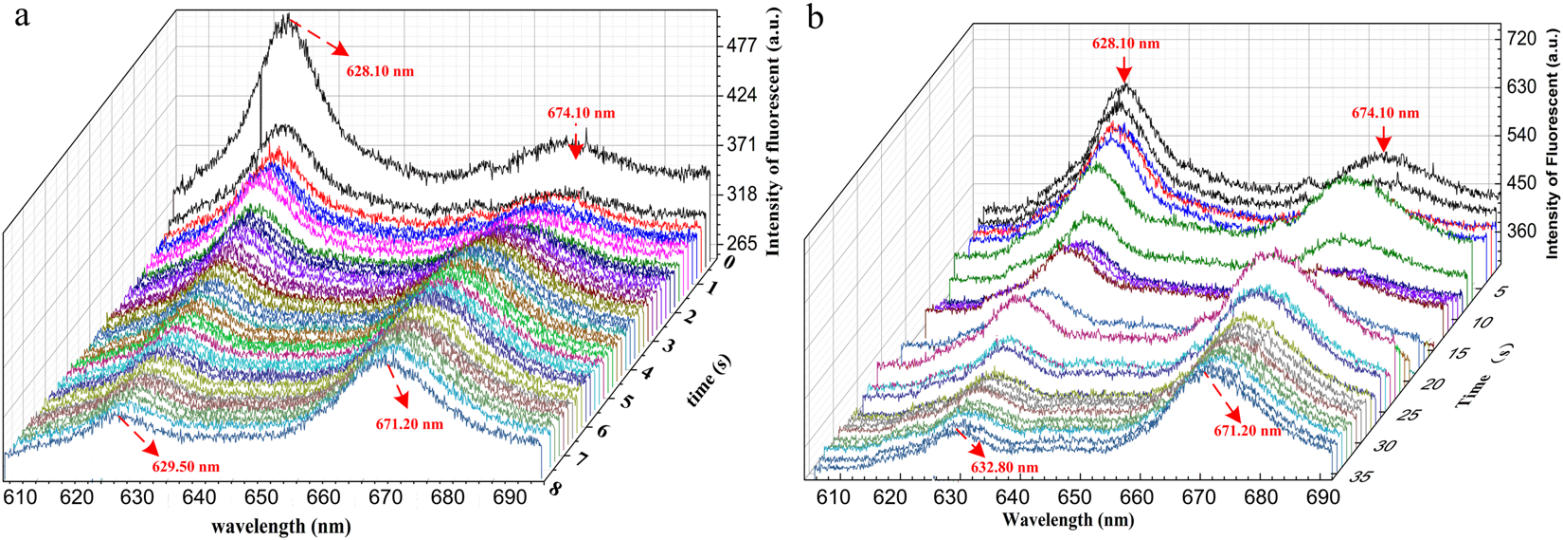
Monitoring the Fe^2+^ and Fe^3+^ combination with Protoporphyrin IX. (**a**–**c**) Dynamic reaction process between Fe^2+^ and Protoporphyrin. The X-, Y- and Z- axis are wavelength (nm), reaction time (s) and fluorescence intensity (a.u.), respectivity; (**d**–**f**) Dynamic reaction process between Fe^3+^ and Protoporphyrin.

The dynamic fluorescent spectra during the whole reaction process are illustrated in Fig. 4. For the reaction includes Fe^2+^, it is obvious that the fluorescence intensity of central wavelength at 629.50 nm reduces gradually for 10 s, while the fluorescent intensity of the other peak at 672.10 nm slowly increases. The fluorescent intensity of both peaks tend towards a steady state in the end. Under the same experimental condition, the reaction between Fe^3+^ and Protoporphyrin IX are also demonstrated, which takes a much longer time to become stable. For the experiment with Fe^3+^, drastic fluctuations can be observed for both fluorescent peaks during the first 25 s, which is completely different from the Fe^2+^ reaction. Several statements can be made on the above results. i) Different central wavelength for the left fluorescent peaks are observed for different reactions, i.e., 629.50 nm for the Fe^2+^ compound and 632.80 nm for the Fe^3+^ compound; ii) The reaction time of Fe^2+^ is shorter than Fe^3+^; iii) The fluorescent emission wavelengths of the above reactions are in good accordance with the Hemoglobin samples shown in Fig. 1f. The Fe^2+^ compound has the same central wavelengths with the normal hemoglobin, while the fluorescent peaks of the Fe^3+^ compound coincide with the SCD hemoglobin.

**Figure 5:**
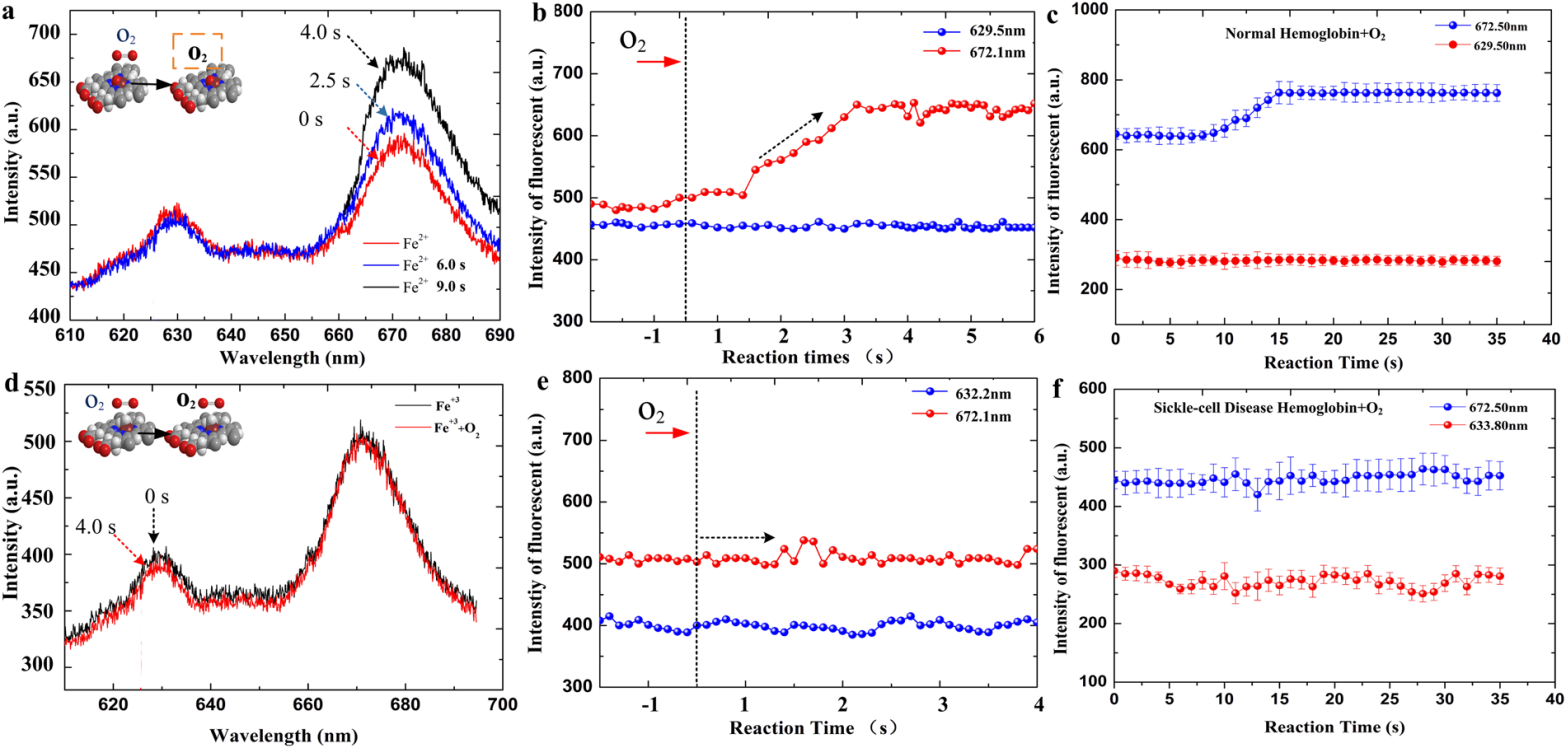
Dynamic oxidation process of the Heme and Metheme, respectively. (**a**) and (**d**) Fluorescent spectra of the Heme and Metheme before and after the oxidation. (**b**) and (**e**) Time dependent variation of the fluorescent peak intensities during the oxidation process. (**c**) and (**f**) Comparison experiments via the Hemoglobin specimen from normal person and SCD patient, which are provided by Ruijin Hospital.

The second experiment is designed to observe the dynamic processes of the reactions between the oxygen gas and the two compounds of the previous experiments. From now on, we will refer to the product of the combination reaction of Protoporphyrin IX and Fe^2+^ as Heme, whilst Metheme is applied to denote the product of the Protoporphyrin IX and Fe^2+^ reaction. In Fig. 5a,d, the fluorescent spectra at different times are recorded, while the time dependent fluorescent intensities of each peak are also plotted in Fig. 5b,e. It is obviously that both the fluorescent spectra and peak intensity varies very little for the Metheme, which indicates that Metheme is difficult to be oxidized, i.e., incapable of binding oxygen. On the other hand, the fluorescent spectra of Heme vary drastically during the oxidation. It is also interesting to note that the fluorescent peak at 672.1 nm increases gradually till the intensity finally reaches a stable value, while the fluorescent peak at 629.5 nm remains unchanged. Comparing the above phenomenon with the Hemoglobin specimen obtained from Ruijin hospital, it is clear that normal Hemoglobin specimen displays a very similar pattern with the Heme product, and the SCD patient specimen resembles the Metheme product. Inner connect between the Heme and normal Hemoglobin must exists based on the above experimental observation, while same remarks can also be applied to the Metheme and SCD Hemoglobin.

## Conclusions

The investigations on SCD are urgent and of significant importance to reduce the children mortality in low resource area. This paper adopts a high sensitivity optofluidic resonator based on a metallic cladding waveguide structure. The enhanced fluorescent effect enables the usage of specimen of very low concentration, while real time detection is also available due to the short switching time. The reactions of Protoporphyrin IX with Fe^2+^ and Fe^3+^ are studied in details, while the dynamic oxidation processes of their products are also researched. Comparison experiments are also carried out via bio-specimen provided by hospital, and a possible hypothesis on the pathogenetic factor of SCD is also proposed, that the heme in the blood of normal person is replaced by the Metheme in the blood of SCD patients.

### Ethics

All methods were carried out in accordance with relevant guidelines and regulations. And all experimental protocols were approved by National Health and Family Planning Commission of the People’s Republic China and Shanghai Jiaotong University. And informed consent was obtained from all subjects.

## Electronic supplementary material

Supplementary information
