# Supplementary material for: A possible pathogenetic factor of sickle-cell disease based on fluorescent analysis via an optofluidic resonator
Source: Sci Rep. 2017 Jun 9;7:3174. doi: 10.1038/s41598-017-03634-8 (PMC5466609; doi:10.1038/s41598-017-03634-8)
Supplement: Supplementary file 1 — Supplementary information [file 41598_2017_3634_MOESM1_ESM.docx]

**Supplementary information for article:**

**A possible pathogenetic factor of sickle-cell disease based on fluorescent analysis via an optofluidic resonator**

Hailang Dai^1,2^, Cheng Yin^1,3^, Xiaona Ye^1,2^, Bei Jiang^1,2^, Maowu Ran^4^, Zhuangqi Cao^1^, Xianfeng Chen^1,2,*^

^1^The State Key Laboratory on Fiber Optic Local Area Communication Networks and Advanced Optical Communication Systems, Department of Physics and Astronomy, Shanghai JiaoTong University, Shanghai 200240, China

^2^Collaborative Innovation Center of IFSA (CICIFSA), Shanghai Jiao Tong University, Shanghai 200240, China

^3^Jiangsu Key Laboratory of Power Transmission and Distribution Equipment Technology, Hohai University, Changzhou 213022, China

^4^Department of Physics and Electronic Science, Tongren University, Tongren, China;

*e-mail: [xfchen@sjtu.edu.cn](mailto:xfchen@sjtu.edu.cn);

This document contains supplementary information to the manuscript, where the quality factors Q, the spontaneous emission rate enhancement ration $\eta$ of the hollow-core metal-cladding optofluidic resonator are discussed in detail in the first part. In the second part, we discuss the issue of fluorescence quenching.

The ultrahigh order modes (UOMs) is excited in guiding layer of hollow-core metal-cladding optofluidic resonator. The UOMS has three properties including high-power density^1^, high-quantity factor^2^ and high-sensitivity^3^. The high-power density provides a combination reaction condition which gives energy to promote the chemical bond and the formation of new compounds. The high-quantity factor enhances fluorescence intensity at low-concentration sample in which the number of molecules can approach single molecule. The high-sensitivity can develop detection of sample composition in the hollow-core of optofluidic resonator by the whole process of chemical reaction.

**

**

Supplementary 1. (a) and (b) are structure of optofluidic resonator; (c) A large number of cavity modes are excited in optofluidic resonator HCMW guiding layer, and descript theory of enhancement fluorescent intensity,$\eta$is represented enhance ratio by HCMW; (d) The four-level energy system of PpIX, the$\omega_{03}$is excitation frequency and spontaneous emission is$\omega_{21}$. The nonradioactive decay is very fast (~ps) and 2-level transform to 1-level need ns time, and collisional quenching cannot occur, due to the concentration of sample solution is less than 10^-13^g/mol.

Supplementary 1 (a) and (b), it has detailed description of the optofluidic resonator structure. When exciting light illuminate the top layer at coupling angles, UOMs can simulate more than 1000 modes constrained in the guide layer by varying the incident angle, induced characteristics of high Q-factor ($Q\text{=}\frac{\lambda}{\Delta\lambda}\text{=}\frac{\text{473nm}}{0.0043\text{nm}}\approx10^{5}$) and high power density. A large number of cavity modes have been excited in hollow-core metal-cladding optofluidic resonator^4^, and UOMs with FWHM less than 0.01nm. As shown in figure 3(a) Filling with samples solution in the hollow core of hollow-core metal-cladding optofluidic resonator, has been used as gain material which has~30nm gain bandwidth.

The UOMs observed via direct incidence of a collimated beam on the chip surface clearly supports the existence of the inter-mode coupling mechanism. The inter-mode coupling refers to the fact that energy of a specific high order mode can be easily transferred to the adjacent modes, due to the high mode density and the small variation between the wave vectors. Another way to understand this feature is that when incident light hits the chip surface, its energy is coupled and stored in the guiding layer, then the energy is leaked out of the waveguide chip through all the possible channels. In this process, the series of reflection cones are formed. To sum up, the energy coupled in the guiding layer can be shifted from one mode to another mode without any outside assistance.

Owing to the inter-mode coupling, the spontaneous emission rate enhancement ration $\eta$ is different from case where only one cavity mode overlaps the gain bandwidth of the laser medium. Suppose $N$ cavity modes overlap the gain bandwidth, and we ignore the small difference between different cavity modes HMFW, there is

$\eta=\left[\sum_{i=1}^{N} \rho_{c}(v_{i})P(v_{i})\Delta\rho_{c}\right]/\left[\rho_{f}(v_{0})P(v_{0})\Delta P\right]$ (1)

where $\rho_{c}(v)\left[\rho_{f}(v)\right]$ is the mode density for photons with (without) the cavity, and $P(v)$ denotes the transition rate. $\Delta P$ is the FWHM of $P(v)$, and $\Delta\rho_{c}$ is the FWHM of the resonant cavity mode. The mode density of each UOM is very high due to the field enhancement effect, on the other hand, the number of the UOMs in the fluorescence region of the dye material is extremely high. Take R6G as an example, the reflection spectrum of the simplified model is plotted in the inset of Fig. 3(c), which shows the UOMs is so dense that the value of $N$is about several hundred.

Low-concentration of sample solution (10^-16^g/ml) and micro-volume of solution injection (0.1mm^3^) has been used to research SCD how to produce anoxia by molecule structure, as a result of the fluorescence intensity has been enhanced 45 times more than without UOMs in cavity. Approach hundreds of molecules or even a single-molecule to analyze Fe^2+^ and Fe^3+^ combination with Protoporphyrin IX and monitor whole process of compound catch oxygen atom based on the fluorescence intensity enhancement.

**References**

1. Y. Wang, Z.Q. Cao, Y. T.Yu, et al. Enhancement of superprism effect based on the strong dispersion effect of ultrahigh-order modes, *Opt. Lett.*, **33,**1276 (2008).
2. [Jingjing Sun](http://scitation.aip.org/content/contributor/AU0973406;jsessionid=DAPyK6JXrNCBiym9KbyimCRg.x-aip-live-06), [Xianping Wang](http://scitation.aip.org/content/contributor/AU0973408;jsessionid=DAPyK6JXrNCBiym9KbyimCRg.x-aip-live-06), [Cheng Yin](http://scitation.aip.org/content/contributor/AU0973409;jsessionid=DAPyK6JXrNCBiym9KbyimCRg.x-aip-live-06), [Pingping Xiao](http://scitation.aip.org/content/contributor/AU0931180;jsessionid=DAPyK6JXrNCBiym9KbyimCRg.x-aip-live-06), [Honggen Li](http://scitation.aip.org/content/contributor/AU0973410;jsessionid=DAPyK6JXrNCBiym9KbyimCRg.x-aip-live-06) and [Zhuangqi Cao](http://scitation.aip.org/content/contributor/AU0363089;jsessionid=DAPyK6JXrNCBiym9KbyimCRg.x-aip-live-06), Optical transduction of *E. Coli* O157:H7 concentration by using the enhanced Goos-Hänchen shift, *J. Appl. Phys.* **112,** 083104 (2012).
3. Yuanlin Zheng, Zhuangqi Cao, and Xianfeng Chen, [Conical reflection of light during free-space coupling into a symmetrical metal-cladding waveguide](http://olab.physics.sjtu.edu.cn/papers/2013/YL_Zheng_josaa-30-9-1901.pdf), *Journal of the Optical Society of America A*, **30**, 9, 1901-1904 (2013).
4. Yuan, W., Yin, C. *et al.* Wideband slow light assisted by ultrahigh-order modes. *Journal of the Optical Society of America B*. 2011; **28(5):**968–971.
